# Directed Differentiation of Human Pluripotent Stem Cells towards Corneal Endothelial-Like Cells under Defined Conditions

**DOI:** 10.3390/cells10020331

**Published:** 2021-02-05

**Authors:** Pyry Grönroos, Tanja Ilmarinen, Heli Skottman

**Affiliations:** Faculty of Medicine and Health Technology, Tampere University, Arvo Ylpön Katu 34, 33520 Tampere, Finland; pyry.gronroos@tuni.fi (P.G.); tanja.ilmarinen@tuni.fi (T.I.)

**Keywords:** corneal endothelial cells, human pluripotent stem cells, neural crest cells, differentiation, small molecule, retinoic acid

## Abstract

The most crucial function of corneal endothelial cells (CEnCs) is to maintain optical transparency by transporting excess fluid out of stroma. Unfortunately, CEnCs are not able to proliferate in vivo in the case of trauma or dystrophy. Visually impaired patients with corneal endothelial deficiencies that are waiting for transplantation due to massive global shortage of cadaveric corneal transplants are in a great need of help. In this study, our goal was to develop a defined, clinically applicable protocol for direct differentiation of CEnCs from human pluripotent stem cells (hPSCs). To produce feeder-free hPSC-CEnCs, we used small molecule induction with transforming growth factor (TGF) beta receptor inhibitor SB431542, GSK-3-specific inhibitor CHIR99021 and retinoic acid to guide differentiation through the neural crest and periocular mesenchyme (POM). Cells were characterized by the morphology and expression of human (h)CEnC markers with immunocytochemistry and RT-qPCR. After one week of induction, we observed the upregulation of POM markers paired-like homeodomain transcription factor 2 (PITX2) and Forkhead box C1 (FOXC1) and polygonal-shaped cells expressing CEnC-associated markers Zona Occludens-1 (ZO-1), sodium-potassium (Na^+^/K^+^)-ATPase, CD166, sodium bicarbonate cotransporter 1 (SLC4A4), aquaporin 1 (AQP1) and N-cadherin (NCAD). Furthermore, we showed that retinoic acid induced a dome formation in the cell culture, with a possible indication of fluid transport by the differentiated cells. Thus, we successfully generated CEnC-like cells from hPSCs with a defined, simple and fast differentiation method.

## 1. Introduction

The corneal endothelium (CEn) is a monolayer of cells on the posterior surface of the cornea that transports water out of the stroma to maintain the cornea transparent [1,2]. Human corneal endothelial cells (hCEnC) are highly polarized flat cells with tightly hexagonal apical surfaces. However, their basal surface is irregular, as they lie on an amorphous collagenous membrane called Descemet’s membrane (DM) [2]. To sustain relatively hydrated cornea, the CEn constantly keeps up the “Pump-Leak” mechanism, where active transport properties such as the sodium-potassium pump (Na^+^/K^+^ ATPase) represents the “Pump” and the corneal swelling pressure presents the “Leak”. All of this is regulated by the barrier properties of tight junction proteins such as Zona Occludens-1 (ZO-1) [3,4]. Mature CEn does not normally replicate in vivo enough to replace dead or damaged cells, since the cell cycle of the hCEnCs is arrested in the G1 phase [4]. The number of hCEnCs gradually decreases with age but, it can also decline dramatically due to endothelial damage caused by trauma or disease, such as Fuchs’ dystrophy, leading to corneal swelling and, eventually, to blindness [5]. Furthermore, damage can be caused by surgical complications, e.g., after intraocular lens implantation [6,7]. Despite that the majority (~80%) of all corneal blindness cases are avoidable and reversible, corneal transplantation is still often needed. To date, keratoplasty is the most performed allogenic transplant worldwide, with a high success rate (90–95%) [8]. Many corneal transplants (185,000) were performed worldwide in 2012, and most of them (39%) were for patients suffering from Fuchs’ dystrophy. Currently, cadaveric donor cornea transplantation using Descemet membrane endothelial keratoplasty (DMEK) or Descemet stripping with automated endothelial keratoplasty (DSAEK) are the preferred therapies for treating the loss of hCEnCs [9]. Unfortunately, the demand of global corneal transplantation was estimated at 12.7 million [10]. Despite the increase in cornea donations in recent years (+1.9% change from 2018 to 2019), there is still huge need for corneal transplants worldwide [8,11]. Extensive methodological development has been made with the in vitro expansion methods for primary cells [12]. However, the quality of cultured CEnCs is largely dependent on donor age; thus, other cell sources totally independent from donor cornea are more desirable. Renewable, expandable and bankable human pluripotent stem cells (hPSC), including embryonic stem cells (hESC) and induced pluripotent stem cells (hiPSC), are becoming attractive sources for the stem cell-based therapy of diseased or wounded corneal layers, including CEnCs [13].

The embryonic development pathway for the human Cen is still not fully known. Previous research has shown that, during eye development, hCEnCs are derived from neural crest cells (NCC)—specifically, from periocular mesenchyme (POM) [14]. Several molecular markers have been used to analyze NCC and POM. For example, transcription factors such as activating enhancer binding protein 2 alpha (AP2α) and transcription factor SOX10 have been used to identify NCCs [15,16] and paired-like homeodomain transcription factor 2 (PITX2) and Forkhead box C1 (FOXC1) to identify POM cells [17]. It is widely known that transforming growth factor beta (TGF-β), WNT and bone morphogenetic (BMP) signaling pathways play crucial roles in NCC and CEnC induction, and thus, many research groups have capitalized on this knowledge in their differentiation protocols [14,16,18,19,20,21]. Thus, over the years, protocols to differentiate hCEnCs from hPSCs have improved significantly, but to our best knowledge, none of the developed protocols have reached clinical grade cell production. Previously, hCEnC-like cells have been successfully differentiated from hPSCs, e.g., by using the bovine CEnC conditional medium [22] or a coculture with human corneal stroma cells and lens epithelial cell-conditioned medium [23]. Chen et al. first used all-trans retinoic acid (RA) to derive NCCs out from mouse embryoid bodies (EB), followed by lens epithelial cell-conditioned medium to differentiate mCEnC-like cells [24]. Steps towards more defined protocols were taken by McCabe et al., who introduced the hCEnC protocol by using Dual Smad inhibition. In their study, NCCs were first derived from hESCs by a TGF beta inhibitor (SB431542) and Noggin, and then, cells were switched to a medium supplemented with a hCEnC induction cocktail, including platelet-derived growth factor B (PDGF-BB), WNT inhibitor/activator Dickopf-related protein 2 (DKK-2) and basic fibroblast growth factor (bFGF) [19]. More recently, Wagoner et al. produced similar hCEnC-like cells, but instead of noggin, they used CHIR99021, a GSK-3-specific inhibitor that leads to WNT activation during NCC induction [20].

Despite these achievements, many of the protocols still include undefined components (e.g., conditioned medium) and animal-derived materials (e.g., Matrigel) that possibly increase the undesired variability in differentiation and are problematic considering the clinical applications. In this study, our goal was to use a more directed protocol with defined components for the differentiation of hCEnC from both hESCs and hiPSCs, facilitating the method translation to clinical use. As an outcome, we introduced a novel, defined and fast method to produce hCEnC-like cells from hPSCs.

## 2. Materials and Methods

### 2.1. Human PSC Establishment and Characterization

Tampere University, Faculty of Medicine and Health Technology, has the approval of the National Authority Fimea (Dnro FIMEA/2020/003758) to conduct research on human embryos. The research group also had supportive statements from the Ethical Committee of the Pirkanmaa Hospital District to derive, culture and differentiate hESC lines (R05116); to establish and use hiPSC lines in ophthalmic research (R16116) and to use human corneas that are not suitable for transplantation in the research (R11134). No new hESC lines were derived for the research conducted here. The previously established hESC line Regea08/017 and hiPSC line WT001.TAU.bB2 from a healthy donor was used in this study. The derivation and culture of the hESC line Regea08/017 has been previously described [25], and here, the establishment of the hiPSC line WT001.TAU.bB2 was shortly described. Peripheral blood monocytes (PBMC) were isolated from 20 mL of healthy donor blood sample, and cells were reprogrammed into human iPSCs using an integration-free CytoTune-iPS Sendai Reprogramming kit (Thermo Fisher Scientific, Waltham, MA, USA) in feeder independent culture conditions according to the protocols provided by the manufacturer. After the manual selection of several clones, the removal of the reprogramming vectors was confirmed, and the further expansion of the hiPSCs (from passage 4 onwards) and cryopreservation were conducted to establish adequate master and working cell banks for research purposes. For that, the feeder-free culture was done as previously described in Hongisto et al. [26] using Corning^®^ CellBIND^®^ 24-well culture plates (Corning, Corning, New York, NY, USA) coated with human recombinant laminin-521 (LN521™, Biolamina, Sundbyberg, Sweden) and Essential 8™ Flex Medium (E8, Thermo Fisher Scientific) supplemented with Essential 8 Flex supplement (50×) and 50-U/mL Penicillin-Streptomycin (Gibco, Thermo Fisher Scientific). The hPSC pluripotency was verified by observing their spontaneous differentiation as embryoid bodies, followed by immunofluorescence labeling for derivative cells of the three embryonic germ layers, as described previously [26]. For the experiments, the quality of hPSCs were continuously monitored for attachment, growth and morphology with a Nikon Eclipse TE2000-S phase contrast microscope (Nikon Instruments Europe B.V. Amstelveen, The Netherlands), and prior to differentiations, the hPSCs were thoroughly characterized (e.g., OCT-3/4, SSEA-3, SSEA-4, TRA-1-81 and LIN28); the normal karyotype was conformed (G-banding service by Fimlab Laboratoriot Oy Ltd., Tampere, Finland) and cells were tested for mycoplasma negativity with Venor^®^GeM Classic (Minerva biolabs, Berlin, Germany) (not shown).

### 2.2. Differentiation of hPSCs into NCCs and Further to CEnC-Like Cells

As described above, the hPSC maintenance was performed on LN521, which was in addition to LN511, a major laminin form present in adult human Descemet’s membranes [27]. Therefore, also, hPSC differentiation was carried out on LN521 to minimize the number of different components in the protocols. First, NCCs were differentiated from hPSCs based on modified protocol from Tchieu et al., as summarized in Supplementary Figure S1a [16]. Briefly, hPSCs were seeded on LN521™-coated CellBind 12 or 24-well plates (Corning, Corning, New York, NY, USA) at 75,000–200,000 cells/cm^2^. The cells were cultured in E8 medium for 24 h. For initiation of the differentiation, a serum=free basal medium consisting of KnockOut Dulbecco’s Modified Eagle Medium (KO-DMEM), 15% KnockOut serum replacement, 2-mM GlutaMax-I, 0.1-mM 2-mercaptoethanol, 50-U/mL penicillin/streptomycin (all from Thermo Fisher Scientific, Waltham, MA, USA), 1% nonessential amino acids (Thermo Fisher Scientific, Sigma-Aldrich, St. Louis, MO, USA) were used. On day 0 of differentiation, E8 medium was replaced with induction medium consisting of the above-mentioned basal medium supplemented with 500-nM bone morphogenetic (BMP) pathway inhibitor LDN193189 (LDN; Sigma-Aldrich) and 10-µM TGF-β inhibitor SB431542 (SB; Stemcell Technologies, Vancouver, BC, Canada). On day 1, 3-µM GSK3 inhibitor/WNT pathway activator CHIR99021 (CHIR; Stemcell Technologies) was added with 10-µM SB and 500-nM LDN. From day 2 onwards, the medium was supplemented with 10-µM SB and 3-µM CHIR. For the N2 medium experiments, the cells were differentiated either with N2 (control, Figure S1a) or without N2 (Figure S1b). In the control culture (Figure S1a), the basal medium was gradually changed to N2 medium during days 4–10 of the differentiation. The N2 content of the whole medium at day 4 was changed to 25%, day 6 to 50%, day 8 to 75% and day 10 to 100%. In the experiment culture (Figure S1b), the N2 medium was discarded completely, and the differentiation was carried out in the basal medium throughout, supplemented as described above. For experiments without LDN and N2 medium (Figure S1c), hPSCs were seeded at 30,000–60,000 cells/cm2 on LN521™, and induction was started on day 0 in the basal medium supplemented with 4-µM CHIR and 10-µM SB throughout.

**Figure 1:**
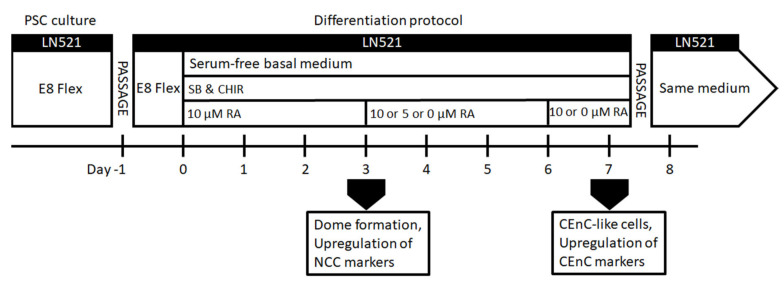
Schematic presentation of the human pluripotent stem cell (hPSC)-CEnC differentiation protocol. Abbreviations: LN521 = human recombinant laminin 521, E8 Flex = Essential 8 Flex Medium, SB = activin/ bone morphogenetic (BMP)/transforming growth factor beta (TGF-β) pathway inhibitor SB431542, CHIR = GSK-3 inhibitor and WNT pathway activator CHIR99021, RA = retinoic acid, NCC = neural crest cell and CEnC = corneal endothelial cell.

For enhancing the differentiation towards POM and CEnCs (Figure 1), hPSCs were seeded on LN521™-coated CellBind 6 or 12-well plates (Corning, Corning, New York, NY, USA) at 10,000–60,000 cells/cm^2^. The cells were cultured in E8 medium for 24 h. On day 0 of the differentiation, E8 medium was replaced with the basal medium supplemented with 10-µM SB, 4-µM CHIR and 10-µM retinoic acid (RA; Sigma-Aldrich). RA was either kept throughout the differentiation, or the concentration was lowered to 5 µM during days 3–6 and then removed or RA was removed completely already after day 3 (Figure 1). After day 7, CEnC-like cells formed, and they were harvested for analyses or further passage into LN521-coated well plates or chamber slides (Thermo Fisher Scientific, Waltham, MA, USA) in the same medium. CEnC-like cells were passaged using the TrypLE Select (Thermo Fisher Scientific) dissociation enzyme and incubated for 3–6 min at 37 °C. Subsequently, the CEnC-like cells were detached gently by trituration, filtered through a 40-µm cell strainer (Thermo Fisher Scientific, Waltham, MA, USA) and centrifuged for 5 min at 300× *g*. Phase contrast light microscope Nikon Eclipse TE2000-S with a DS-Fi1 camera (Nikon Corp., Tokyo, Japan) was used to capture images of the cell morphology.

### 2.3. Harvesting of Primary hCEnCs for Cell Culturing

A donor human cornea of a young adult, which was not suitable for transplantation due to hepatitis infection of the donor, was used for harvesting CenCs. Cornea was inserted CenC side-up on a cutting block of Barron Marking Corneal Donor Punch (Katena, Parsippany, NJ, USA). To form a well, the seating ring was placed on the top of cornea, which was filled with TrypLE Select and incubated for 15 min at 37 °C. Subsequently, the cells were detached by trituration or scratching and seeded on collagen IV (Sigma-Aldrich) and LN521™-coated CellBind 24-well plates (Corning, Corning, New York, NY, USA). The primary CenCs were cultured until confluency (3 weeks) in a medium consisting of DMEM/F12, 10% fetal bovine serum, 2-mM GlutaMax-I, 50-U/mL penicillin/streptomycin (all from Thermo Fisher Scientific, Waltham, MA, USA), 2-ng/mL bFGF (Peprotech, Rocky Hill, NJ, USA) and 10-µM ROCK inhibitor Y27632 (Stemcell Technologies). Medium was changed twice a week.

### 2.4. Immunocytochemistry

The cells were fixed with 4% paraformaldehyde (PFA, Sigma-Aldrich) for 15 min. Next, the cells were permeabilized for 10 min with 0.1% Triton X-100 (Sigma-Aldrich), followed by blocking with 3% bovine serum albumin (BSA) for 1 h. Then, the cells were first incubated with 1:400 SOX10 (Santa-Cruz, Santa Cruz, CA, USA, sc-365692) 1:400 activating enhancer binding protein 2 alpha (AP2α; Thermo Fisher Scientific, MA1-872), 1:400 paired box protein 6 (PAX6; Sigma-Aldrich, HPA030775), 1:400 zona occludens-1 (ZO-1; Thermo Fisher Scientific, 61-7300), 1:200 anti-alpha 1 sodium potassium ATPase (Na^+^/K^+^-ATPase, Abcam, Cambridge, UK, ab7671), 1:400 CD166 (BD Biosciences, 559260 and Abcam, ab109215), 1:400 N-cadherin (NCAD; Abcam, ab18203 and Sigma-Aldrich, C2542), 1:400 paired-like homeodomain transcription factor 2 (PITX2; Thermo Fisher Scientific, PA5-11479), 1:400 Forkhead box C1 (FOXC1; Abcam, ab227977) and 1:400 octamer binding transcription factor (OCT) ¾ (R&D Systems, Minneapolis, MN, USA, AF1759) primary antibodies overnight at 4 °C. The cells were next treated with 1:800 donkey anti-rabbit immunoglobulin G (IgG) secondary antibody, Alexa Fluor 488, 1:800 donkey anti-rabbit IgG secondary antibody, Alexa Fluor 568, 1:800 donkey anti-mouse IgG secondary antibody, Alexa Fluor 488, 1:800 donkey anti-mouse IgG secondary antibody, Alexa Fluor 568, 1:800 donkey anti-goat IgG secondary antibody, Alexa Fluor 488, 1:800 donkey anti-goat IgG secondary antibody or Alexa Fluor 568 (all from Thermo Fisher Scientific, Waltham, MA, USA), according to the host of the primary antibody for 1 h at room temperature. The nuclei were counterstained with 4′,6-diamidine-2′-phenylindole dihydrochloride (DAPI; Vector Laboratories, Peterborough, UK) or Hoechst 33342 (Thermo Fisher Scientific, Waltham, MA, USA). The images of mounted cells were captured using a fluorescence microscope (Olympus IX51; Olympus, Tokyo, Japan) or confocal microscope (Zeiss LSM 700, Zeiss LSM 800, Carl Zeiss AG, Oberkochen, Germany) and prepared using image editing software (Adobe Photoshop CC 2018; Adobe Inc., San Jose, CA, USA)

### 2.5. Immunohistochemistry

A human donor cornea was fixed with 4% PFA for 4 h at room temperature (RT) and embedded into a paraffin block. The block was sectioned with microtome. Sections were attached on glass slides at 60 °C for 90 min. Then, they were deparaffinated with xylene for 5, 4, 3 and 1 min, then 2 × 4 min 99.9% ethanol, 2 × 3 min 96% ethanol and 3 min 70% ethanol. Finally, sections were hydrated in water. Antigen retrieval was concluded with boiling 10-mM sodium citrate and 0.05% Tween 20 in pH 6 for 12 min. Samples were treated with blocking buffer consisting of 10% donkey serum in TBS and 5% bovine serum albumin (BSA) for 1 h at 37 °C in a moisture chamber. Then, the sections were incubated with 1:50 NCAD (Sigma-Aldrich, C2542), 1:50 Na^+^/K^+^-ATPase (Abcam, ab7671) and 1:50 CD166 (Abcam, ab109215) primary antibodies in blocking buffer overnight at +4 °C in a moisture chamber. Then, they were incubated in 1:200 donkey anti-rabbit IgG secondary antibody, Alexa Fluor 488 for CD166 and 1:200 donkey anti-mouse IgG secondary antibody, Alexa Fluor 568 for NCAD and Na^+^/K^+^-ATPase for 1 h in a RT moisture chamber. Sections were mounted, and nuclei were counterstained with ProLong Gold Antifade Mountant with DAPI (Thermo Fisher Scientific, Waltham, MA, USA). The images of mounted cells were captured using a confocal microscope (Zeiss LSM 700, Carl Zeiss AG, Oberkochen, Germany) and prepared using image editing software (Adobe Photoshop CC 2018; Adobe Inc.).

### 2.6. RNA Extraction and RT-qPCR

Total RNA was extracted from undifferentiated hPSCs (d0) and from 3 time points during hCEnC induction (d3, d6 and d9) with a Rneasy Mini Kit Plus (Qiagen, Hilden, Germany) or using TRI reagent (Sigma-Aldrich). RNA concentration of each sample was determined using NanoDrop-1000 spectrophotometer (NanoDrop Technologies, Wilmington, DE, USA). RNA was purified from endogenous DNA using Dnase I (Thermo Fisher Scientific, Waltham, MA, USA). From each RNA sample, 400 ng were used to synthesize complementary DNA (cDNA) using the High-Capacity cDNA RT kit (Applied Biosystems, Foster City, CA, USA). The resulting cDNA samples were analyzed with qPCR using sequence-specific TaqMan Gene Expression Assays (Thermo Fisher Scientific, Waltham, MA, USA) for OCT4 (Hs00999632_g1), PITX2 (Hs01553179_m1), aquaporin 1 (AQP1) (Hs01028916_m1), ATPase subunit alpha 1 (ATP1A1) (Hs00167556_m1), Cadherin 2 (Hs00983056_m1), SLC4A4 (Hs00186798_m1) and FOXC1 (Hs00559473_s1). All samples (*n* = 1/condition) were run as triplicate reactions with the 7300 Real-Time PCR system (Applied Biosystems). Results were analyzed with the 7300 System SDS Software (Applied Biosystems) and Microsoft Excel. Based on the cycle threshold (C_T_) values given by the software, the relative quantification of each gene was calculated by applying the −2^ΔΔCt^ method [28]. Results were normalized to Glyceraldehyde 3-phosphate dehydrogenase (GAPDH) (Hs99999905_m1), with the undifferentiated hPSCs as the calibrator to determine the relative quantities of gene expression in each sample.

### 2.7. Cell Density Calculation

The density of the hPSC-CenC-like cells was calculated manually from ZO-1 immunofluorescence images using image editing software (Adobe Photoshop CC 2018; Adobe Inc.).

## 3. Results

### 3.1. Induction of NCC from hPSCs Formed Distinct Cell Populations Expressing PAX6, SOX10 or AP2α 

**Figure 2:**
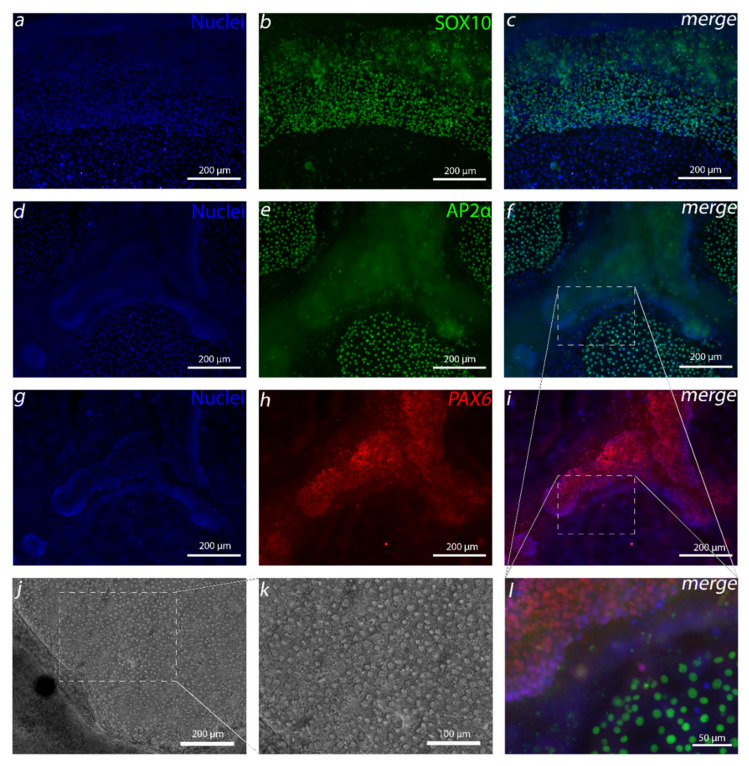
NCC induction resulted in three distinct cell populations. After 14 days of NCC induction, three distinct cell populations were discovered: (**a**–**c**) cells expressing SOX10, (**d**–**f**) activating enhancer binding protein 2 alpha (AP2α) and (**g**–**i**) paired box protein 6 (PAX6). (**l**) AP2α and PAX6 images are merged and enlarged 3× to highlight distinct populations between PAX6 and AP2α-positive cells. (**j**,**k**) Phase contrast light microscope images show the polygonal morphology of the monolayered AP2α-positive cells. Data conducted with the Regea08/017 human embryonic stem cell (hESC) line. Scale bars: (**a**–**j**) 200 µm, (**k**) 100 µm and (**l**) 50 µm.

**Figure 3:**
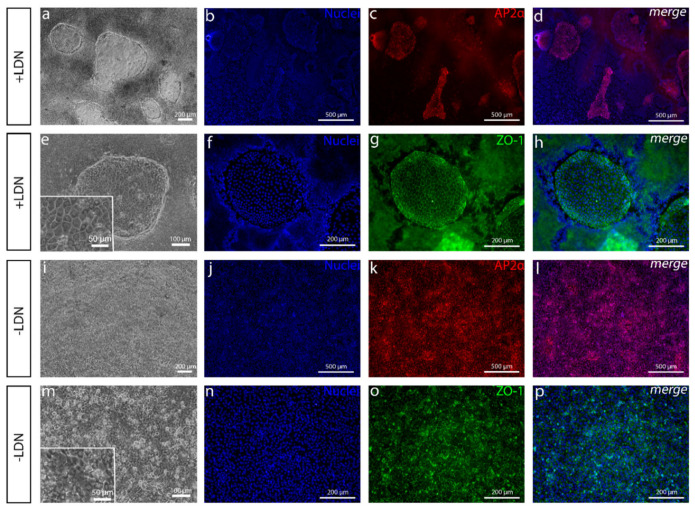
Comparison of cell cultures with or without BMP inhibition. (**a**,**e**–**p**) After 8 days and (**b**–**d**) 13 days of differentiation, (**a**,**e**), phase contrast microscope images and (**b**–**d**,**f**–**h**) immunofluorescence images of cell cultures with pathway inhibitor LDN193189 (LDN) show (**b**–**d**) the AP2α-positive polygonal cell colonies where (**f**–**h**) zona occludens-1 (ZO-1) is localized in tight junctions surrounded by a thick cell mass. (**i**–**p**) Cell cultures without LDN show that (**i**,**m**) the thick cell mass is absent, and (**j**–**l**) AP2α is positive for all the cells. (**n**–**p**) ZO-1 is in tight junctions as well, but the cells are less polygonal. Data conducted with the Regea08/017 hESC line. Scale bars: (**a**,**f**–**i**,**n**–**p**) 200 µm, (**b**–**d**,**j**–**l**) 500 µm, (**e**,**m**) 100 µm and (**e**,**m** magnified images) 50 µm.

We first pursued differentiating NCCs from hPSCs with a serum-free protocol that is relatively simple and short. At first, differentiation was initiated with the method adapted from Tchieu et al. [16]. Following the protocols (Figure S1), the NCC differentiation was based on TGF-β inhibition, BMP inhibition and WNT pathway activation, followed by a gradual change to N2 medium. Adhered LN521-plated hPSCs were induced for two days with 500-nM LDN, continuously with 10-µM SB and, starting from the second day of differentiation, continuously with 3-µM CHIR (Figure S1a). After day 4, the serum-free basal medium was gradually changed to N2 medium for the period of six days. After two weeks of differentiation, the majority of the cell culture was composed of a thick cell mass. The cells in these dense areas were mainly positive for neuroectodermal marker PAX6 (Figure 2g–i,l). There were also spindle-shaped cells mainly bursting out of the edges of the thick cell mass. These cells were positive for NCC marker SOX10 (Figure 2a–c). Furthermore, there was a third distinctive cell population that formed small colonies of monolayered polygonal cells surrounded by the thick cell mass. These cells were positive for another NCC marker, AP2α (Figure 2d–f,l). The AP2α-positive cells also showed cEnC-like characteristics such as polygonal morphology (Figure 3e) with the ZO-1 marker located in the tight junctions (Figure 3f–h), and they were positive for Na^+^/K^+^-ATPase (Figure S2). Next, we aimed to characterize these polygonal AP2α-positive cells further and pursued to develop a culture method to increase the yield of these cells. 

### 3.2. Discard of BMP Inhibition from the NCC-Induction Protocol Increased the Number of AP2α-Positive Cells

**Figure 4:**
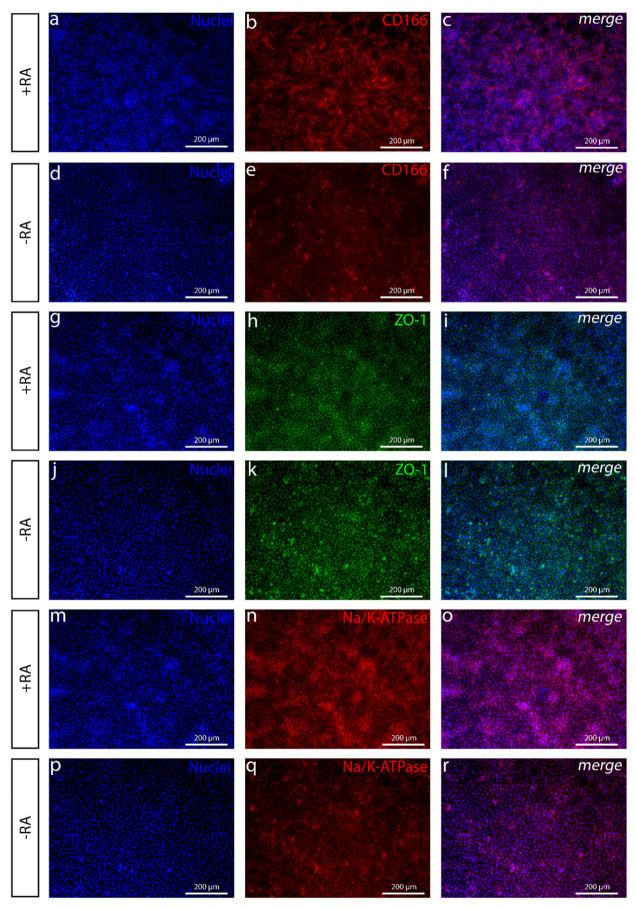
Immunofluorescence images of common CEnC markers with or without RA shown for the Regea08/017 hESC line. After day 11 of differentiation, (**a**–**f**) CD166 is substantially stronger when (**a**–**c**) RA is used (+RA) in the induction compared to when (**d**–**f**) RA is absent (−RA). (**g**–**l**) Tight junction marker ZO-1 shows more polygonal and tightly packed cells (**g**–**i**) with +RA compared to the cell culture (**j**–**l**) without −RA. (**m**–**r**) Na^+^/K^+^-ATPase is important for the pump activity, and the expression was higher in (**m**–**o**) the cell culture with RA compared to (**p**–**r**) the cell culture without RA. Scale bar (**a**–**r**) 200 µm. Corresponding human induced pluripotent stem cell (hiPSC) data is shown in Figure S5.

During the method development, we noticed that the gradually increasing N2 medium did not increase the size or number of the colonies containing polygonal-shaped cells (Figure S3). Hence, it was removed from the protocol. However, increasing the concentration of CHIR to 4 µM showed the enlargement of these cell colonies (Figure S4). Moreover, the highest impact was detected when we discarded LDN from the differentiation protocol (Figure 3). The thick cell mass decreased dramatically (Figure 3a,e,i,m), and the majority of PAX6 and SOX10-positive cells disappeared with it (data not shown), but the number of monolayered AP2α-positive cells increased substantially (Figure 3b–d,j–l). The AP2α-positive cells also showed ZO-1 associated with tight junctions (Figure 3n–p) and other CEnC-like characteristics, including faint CD166 (Figure 4d–f) and Na^+^/K^+^-ATPase (Figure 4p–r) expression in the cell membranes. Despite the positive effects of LDN removal on AP2a expression, the cell morphology was less polygonal and more irregular than with LDN (Figure 3a,e,i,m). At this point, the differentiation protocol included only 10-µM SB and 4-µM CHIR as the induction substances. Next, we pursued modifying the protocol to make the cells differentiate more towards CEnC-like cells.

### 3.3. The Addition of RA Produced POM-Like Structures and Polygonal Cells with Characteristic CEnC Markers

**Figure 5:**
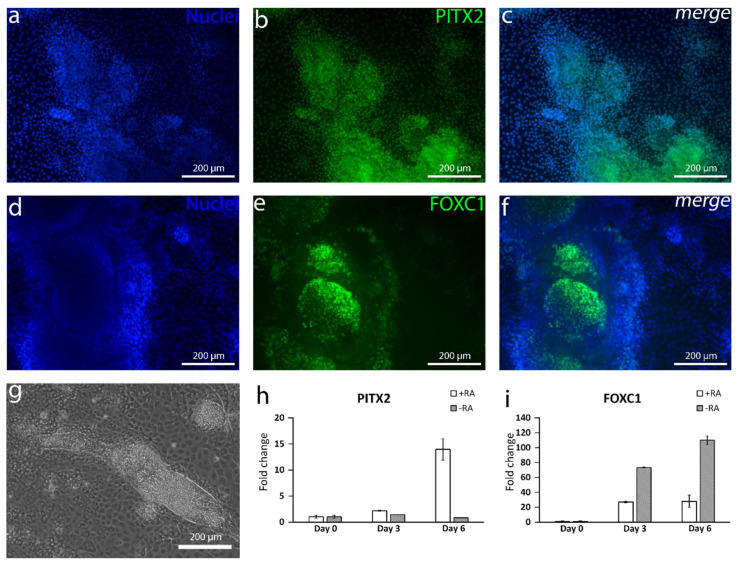
Expression of common periocular mesenchyme (POM) markers in the differentiated cell culture. After 11 days of differentiation, (**a**–**c**) the paired-like homeodomain transcription factor 2 (PITX2) expression is stronger in the thick mesenchyme-like structures. (**d**–**f**) Forkhead box C1 (FOXC1) can be seen in the same structures as well. (**g**) POM-like structures are surrounded by CEnC-like cells. (**h**) RT-qPCR analyses of the differentiating cells with or without retinoic acid (RA) shows an increasing expression of PITX2, especially in +RA but not in −RA. (**i**) The FOXC1 expression increases in both circumstances but more in −RA. Data conducted with the Regea08/017 hESC line. RT-qPCR analyses included three technical replicates. Scale bar (**a**–**g**) 200 µm.

Since the RA gradient has a great impact on the direction where NCCs will further differentiate, it has been shown that the ocular structures demand relatively high RA concentrations in the developing embryo [29]. We thus hypothesized that adding RA to the differentiation protocol would enhance the characteristic CEnC features of the cells. With the addition of 10-µM RA during the first three days of the differentiation protocol, thick islands of mesenchymal-like structures slowly started to appear and were located on the periphery areas of the cell cultures. Interestingly, immunocytochemistry showed that those cells were positive for POM markers FOXC1 (Figure 5a–c) and PITX2 (Figure 5d–f). FOXC1 was often witnessed to be focused at the edge areas of the islands. RT-qPCR results further confirmed the increasing expression of PITX2 with +RA supplementation as compared to −RA (Figure 5h). The expression of FOXC1 increased with both +RA and −RA but more in −RA during the six days of induction (Figure 5i). Interestingly, after the addition of RA, the AP2α-positive cells turned distinctly more towards a CEnC-like morphology, and the cells became more polygonal (Figure 4g–l). The CEnC-like cells were also more densely packed, as demonstrated with the cell counting, i.e., hESCs differentiated with +RA supplementation had 2211 ± 148 (mean ± SD) cells/mm^2^, whereas cells in −RA culture had only 1486 ± 62 cells/mm^2^ (mean ± SD) (data not shown). We also noticed that, at this point, the cell density clearly affected the result. If the cells were plated too scarcely, e.g., under 20,000 cells/cm^2^, the differentiation was not initiated properly. If plated too densely, e.g., over 60,000 cells/cm^2^, the thick mesenchyme-like structures took over the culture, and CEnC-like cell yields were lower. Thus, a plating density between 30,000–50,000 cells/cm^2^ was used. After a week of the differentiation, the lateral membrane of the cells became substantially more positive for CEnC markers CD166 (Figure 4a–f) and Na^+^/K^+^-ATPase (Figure 4m–r) in +RA culture when compared to −RA culture. Both the examined hESC line (Figure 4 and Figure 5) and hiPSC line (Figures S5 and S6) behaved similarly. The preliminary in vitro safety of the differentiated hPSC-CEnCs was also demonstrated by the complete lack of pluripotency marker OCT3/4 (POU5F1) expression analyzed both with immunofluorescence and RT-qPCR analyses (Figure S7a–c,g). In conclusion, with the addition of RA in the differentiation protocol, we managed to produce POM-like structures and, furthermore, polygonal cells with CEnC-like characteristics.

### 3.4. Dome Formation in the Cell Culture with RA Indicates Fluid Transport

**Figure 6:**
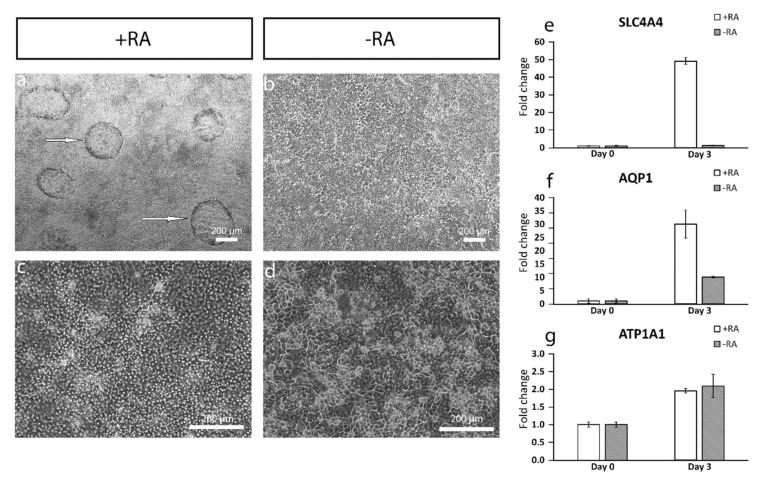
Dome formation was observed already at day 3 of the differentiation. (**a**) White arrows point to the dome structures in the cell culture with RA at day 3. (**b**) Domes are not present in cultures without RA. (**c**) Cells are more polygonal in cell culture with RA at day 10 (RA lowered to 5 µM for days 3–6 and 0 µM after day 6) compared to (**d**) cell culture without RA. Expression of RT-qPCR markers of pump-related genes are increased during the differentiation. (**e**) Strong increase can be seen in the expression of sodium bicarbonate cotransporter 1 (SLC4A4) in +RA but not in the −RA. (**f**) Expression of water channel aquaporin 1 (AQP1) is increased in both +RA and −RA but faster in +RA. (**g**) The increase in the expression of pump protein ATPase subunit alpha 1 (ATP1A1) is moderate. Data conducted with (**a**,**b**) the WT001.TAU.bB2 hiPSC line and (**c**–**g**) Regea08/017 hESC line. RT-qPCR analyses included three technical replicates. Scale bar (**a**–**d**) 200 µm.

Controlled pumping of liquids out of the stroma is the primary function for CEnCs [3,4]. Interestingly, after three days of +RA induction, the cells formed dome-like structures in the cell culture, suggesting plausible fluid transport and barrier-like properties (Figure 6a). Domes were not present in −RA culture (Figure 6b). Furthermore, the cells were more polygonal in the +RA cell culture (Figure 6c) compared to the −RA cell culture (Figure 6d). The RT-qPCR analyses confirmed a strong increase in the expression of electrogenic sodium bicarbonate cotransporter 1 (*SLC4A4*) with +RA supplementation as compared to −RA condition (Figure 6e). Further, the expression of water channel protein aquaporin 1 (*AQP1*) increased in +RA conditions as well (Figure 6f). Na^+^/K^+^-ATPase encoding gene sodium/potassium-transporting ATPase subunit alpha 1 (*ATP1A1*) also slightly increased in expression levels, although there was no difference between +RA and −RA conditions (Figure 6g). Thus, the formation of domes and increased expression of transporters supported the hypothesis that the cells have CEnC-like functional features with +RA supplementation. However, long-term exposure to 10-µM RA caused some of the cells to form big vacuoles and cells with double or more nuclei started to form (Figure S8). Therefore, the amount of RA was lowered after three days of differentiation, and the best results were obtained by lowering the RA concentration to 5 µM for three days and then discarding the RA completely at day 6 (not shown). 

### 3.5. hPSC-CEnCs Considerably Resembles Primary CEnCs and Passaging the Cells Notably Refines the Cell Culture

**Figure 7:**
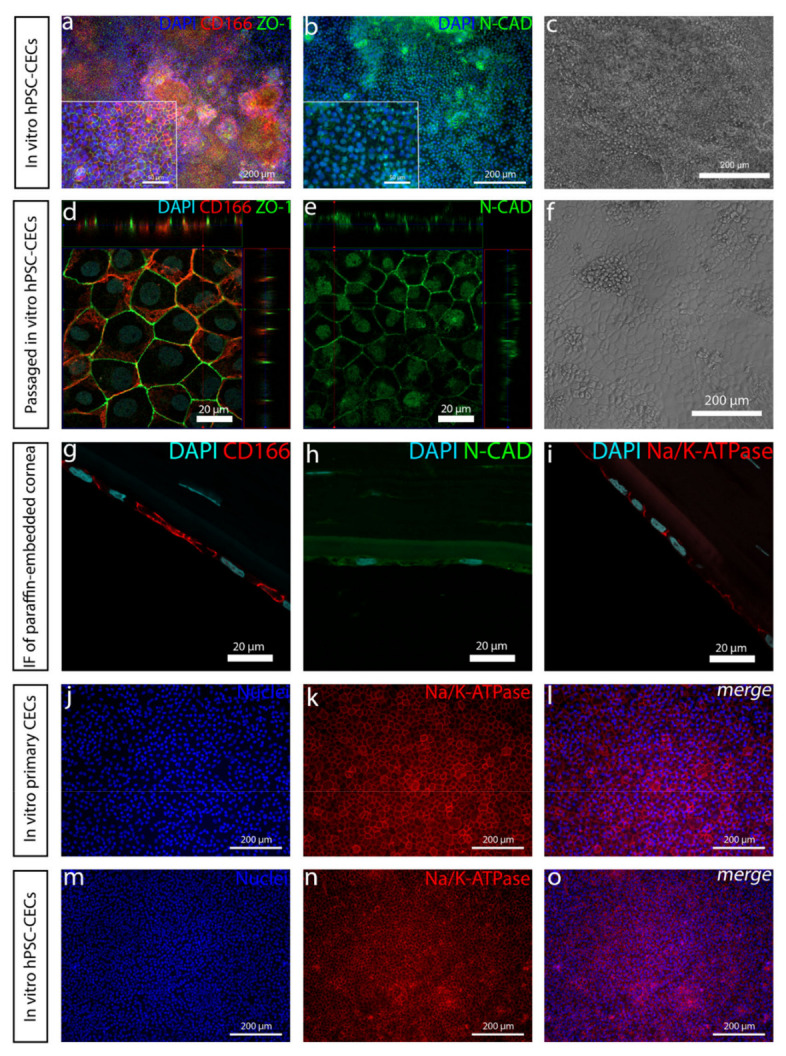
Passaging of the cells refines the cell culture, and the PSC-CEnC-like cells significantly resemble primary CEnC cells. (**a**) Immunofluorescence images of the expression of ZO-1 and CD166 in hPSC-CEnCs at day 8. (**d**) Day 8-passaged hPSC-CEnC-like cells at day 13 and (**g**) primary CEnCs show how the passaging refines the cells and how the differentiated cells resemble primary CEnCs. (**e**) N-cadherin is enhanced in day 8-passaged hPSC-CEnC-like cells at day 13 compared to (**b**) unpassaged day 8 cells. (**c**,**f**) Phase contrast light microscope images show how the cell culture is refined with more hexagonal cells after the passage. (**i**) The expression and localization of Na^+^/K^+^-ATPase are very similar in histological paraffin-embedded endothelium, (**j**–**l**) in vitro primary CEnCs and (**m**–**o**) differentiated PSC-CEnC-like cells at day 8. hPSC-CEnC-like cell data was conducted with the Regea08/017 hESC line. Scale bars: (**a**–**c**,**f**,**j**–**o**) 200 µm, (**a**,**b** magnified images) 50 µm and (**d**,**e**,**g**–**i**) 20 µm.

After a week of the differentiation, the mesenchymal-like structures outcompeted the CEnC-like cells. Passaging the cells considerably refined the cell culture, although some mesenchymal-like cells remained after the passage. Regardless, the cell monolayer kept its polygonal morphology and positivity of ZO-1, CD166 and N-cadherin (Figure 7a–e). While pursuing clinically relevant hPSC-CEnCs, it is crucial to compare the cells with primary CEnCs. When we compared our differentiated hPSC-CEnC-like cells (Figure 7d–f,m–o) with in vitro cultured primary human CEnCs (Figure 7j–l) or histological corneal cross-section samples (Figure 7g–i), we confirmed a clear similarity between these cell types, as demonstrated with the expression of CD166, Na^+^/K^+^-ATPase, N-cadherin and ZO-1 (Figure 7d,e,m–o).

## 4. Discussion

In the complex development of the human cornea, it has been found that several regions of the human eye, including CEn, are derived from NCC-derived POM cells [14,30]. Our first goal was to generate these cells before aiming to differentiate CEnCs. The first attempt to produce NCCs was greatly inspired by Tchieu et al. [16]. The resulting small AP2α-positive colonies of cells surrounded by a thick cell mass that had clear similarities with CEnCs caught our attention. Due to the importance of the AP2 family to CEn development [31], we pursued to find a method to increase the amount of these cells. Surprisingly, discarding BMP inhibition from the protocol had the highest impact on the yield of AP2α-positive cells. BMP activity is known to have effects on NCC differentiation, but the dynamics are poorly understood, and the optimal level of BMP activity is difficult to adjust [32]. Furthermore, in more undefined culture conditions, BMP ligands can be present in used media components, or those can be secreted endogenously by the differentiating side populations, which makes the controlling the in vitro concentration difficult [32,33]. In our case, discarding the exogenous BMP inhibition from the defined differentiation protocol removed the unwanted thick PAX6 positive cell mass, leaving, primarily, the AP2α-positive cells. In our hands, in addition to PAX6 and AP2α, the Tchieu et al. [16]-based protocol produced colonies of SOX10-positive cells that eradicated upon the removal of BMP inhibition and increase of CHIR concentration. SOX10 is a commonly used NCC marker that is regulated by WNT signaling, and it has been shown in *Xenopus* to be expressed more in trunk neural crest (NC) and lesser in cranial NC [34], from which POM and CEn are derived [30]. Interestingly, the findings of Gomez et al. showed that, when differentiating NC from hESCs, CHIR concentrations below 6.5 µM and above 3.5 µM yield no SOX10-positive cells [35]. Thus, the increase of the CHIR concentration from 3 µM to 4 µM in our study might give an explanation why SOX10 cells disappeared from our differentiation cultures as well. This may also explain why our cell cultures without RA were more confluent, containing intact round cells instead of SOX10-positive scattered spindle like cells that Wagoner et al. differentiated with 3-µM CHIR [20].

The capability of producing NCC with TGF-β inhibition and WNT activation and with the further evidence that a relatively high RA concentration guides NCC to form POM [29] convinced us to hypothesize that mixing these agents together would induce the differentiation of CEnCs. RA is an essential morphogen signaling molecule, as well as an important regulator of embryonic development, and the dysregulation of RA levels during embryogenesis has been associated with ocular defects [29]. It has been demonstrated that, during NCC migration, RA activity is the highest within the prosencephalon and developing eye [29,36]. Previous studies have shown that the absence of RA signaling in the eye development downregulates the expression of POM markers *PITX2* and *FOXC1* [37]. Similar results were gained in our study where the addition of RA clearly increased *PITX2* expression during the first days of differentiation. Interestingly, the *FOXC1* expression was higher at the mRNA level without RA supplementation, potentially due to the presence of other NC derivatives than cornea cells [38]. At the protein level, the +RA supplementation did induce the formation of mesenchymal-like structures positive for both PITX2 and FOXC1. 

The addition of RA increased the polygonal morphology of the cells, as well as the expression of important hCEnC makers CD166, Na^+^/K^+^-ATPase and ZO-1, with correct intracellular localization and the formation of dome structures created by the detachment of the cell layer from the culture surface due to the fluid accumulation in between. This suggested fluid transport by the cells that could potentially reflect the pump activity, which was further supported by the increased expression of *AQP1*, *ATP1A1* and *SLC4A4*. Especially, the difference between +RA and −RA conditions regarding the expression of the sodium bicarbonate cotransporter *SLC4A4* was clear, and the RA supplementation had a clear upregulating impact on its expression. The SLC4A4 transporter is crucial for hCEnC, having an important role in maintaining the solute gradients essential for the regulation of water transport from the stroma to the anterior chamber [39]. In the native cornea, the basolateral membrane of CEnCs pump fluids out of the stroma through the Descemet membrane [3], whereas, in the dome structures formed in our in vitro cultures, the potentially active fluid transport appears to be more apical to basal and, thus, directed towards the cell culture plastic. We assumed this was due to the early stage of the maturation, and the cells were not yet fully polarized. Confocal immunofluorescence images of Na^+^/K^+^-ATPase of the cells in the dome structure could confirm this hypothesis, but further methodological development is needed to harvest a proper intact dome structure into immunocytochemistry analyses from cell culture plates. Further functional studies are also needed to address this aspect in more detail to confirm the appropriate pump properties in order to ensure clinical efficacy. During the differentiation method development, we also noticed that prolonged use of RA in the differentiation protocol caused some of the cells to accumulate big intracellular vacuoles, potentially indicating cellular stress (Figure S8). This could be a consequence from too-intense RA induction, which can disrupt the Golgi apparatus [40]. We also noticed that the total removal of RA from the induction phase (during days 3–6) prevented vacuolization, but the amount of dome structures decreased, and the cells lost some of their polygonality. Therefore, finding the balance for the timing and concentration with the RA supplementation is crucial for optimal differentiation.

In comparison to previous publications, the clear difference in our protocol compared to the others is that we only used RA in addition to SB and CHIR, with relatively fast differentiation of the CEnC-like cells. Several other groups used the B27, PDGF-BB, DKK-2 cocktail discovered by McCabe et al. [19] to further differentiate CEnCs from NCCs [19,20,41]. Replacing this cocktail with early RA induction seemed to produce CEnC-like cells with very short induction times needed. Nevertheless, the optimal RA concentration in later stages of the CEn maturation is still to be discovered, together with the further development and in vivo functional assessment of hPSC-CEnCs. 

To conclude, in this study we developed a simple and directed differentiation protocol to derive CEnC-like cells from hPSCs with a small molecule inhibition of TGF-β and induction of WNT combined with RA. Our results demonstrated successfully differentiated hPSC-CEnC-like cells expressing proteins important for CEnCs [2] and with a polygonal, tightly packed morphology. The developed method is fast, feeder-free and uses only defined components. In addition, xeno-free components are available for the final production development for clinical applications, including xeno-free SR (Thermo Fisher Scientific, Waltham, MA, USA). 

## 5. Patents

Tampere University has filed a patent application related to the cell differentiation technology (FI20205857). Based on the Act on the Right in Inventions made at Higher Education Institutions in Finland, all authors employed by Tampere University gave all rights to the university, and thus, the authors declare that they have no competing interests. 

## Data Availability

Not applicable.

## Supplementary Materials

The following are available online at https://www.mdpi.com/2073-4409/10/2/331/s1: Figure S1: Schematic presentation of evolving hPSC-NCC differentiation protocols. Figure S2: The faint expression of Na+/K+-ATPase in AP2α-positive colonies in the NCC induction protocol. Figure S3: N2 medium did not contribute the differentiation at day 14 of the NCC induction protocol. Figure S4: Raising the concentration of CHIR in NCC induction enlarged the AP2α-positive cell colonies. Figure S5: Immunofluorescence images of common CEnC markers with or without RA in the hiPSC line. Figure S6: Expression of common POM markers in the differentiated cell culture in the human iPSC line. Figure S7: The expression of the pluripotency marker decreases totally during the differentiation. Figure S8: Long RA induction makes some of the cells form vacuoles and multiple nuclei.
