# Supplementary material for: Directed Differentiation of Human Pluripotent Stem Cells towards Corneal Endothelial-Like Cells under Defined Conditions
Source: Cells. 2021 Feb 5;10(2):331. doi: 10.3390/cells10020331 (PMC7915025; doi:10.3390/cells10020331)
Supplement: Supplementary file 1 [file cells-10-00331-s001.zip › Captions for supplement figures.docx]

**Captions for supplement figures**

**Figure S1** Schematic presentation of evolving hPSC-NCC differentiation protocols. (A) Control protocol based on Tchieu et al. NCC protocol. (B) Protocol without N2 medium. (C) Protocol without N2 medium and LDN supplement. CHIR concentration was 3 µM in A and B but increased to 4 µM in C. SB was 10 µM and LDN 500 nM in every protocol. Abbreviations: LN521 = human recombinant laminin 521, E8 Flex = Essential 8 Flex Medium, SB = activin/BMP/TGF-β pathway inhibitor SB431542, LDN = bone morphogenetic (BMP) pathway inhibitor LDN193189, CHIR = GSK-3 inhibitor and WNT pathway activator CHIR99021.

**Figure S2** The faint expression of Na^+^/K^+^-ATPase in AP2α-positive colonies in NCC induction protocol. (a-c) Fluorescence images of the NCC differentiation cell culture at day 9 shows Na^+^/K^+^-ATPase expression in AP2α positive cells. Data conducted with Regea 08/017 hESC line. Scale bar (a-c) 200 µm.

**Figure S3** N2 medium did not contribute the differentiation. At day 14 of NCC induction protocol. (a) cell culture without N2 medium showed no change or slightly better AP2α positive cell colonies than (b) with N2 medium. Data conducted with Regea 08/017 hESC line. Scale bar (a-b) 200 µm.

**Figure S4** Raising the concentration of CHIR in NCC induction enlarged the AP2α positive cell colonies. At day 13 of NCC induction protocol, (a) AP2α positive cell colonies in 3 µM CHIR are smaller than in (b) 4 µM CHIR. Data conducted with Regea 08/017 hESC line. Scale bar (a-b) 400 µm.

**Figure S5** Immunofluorescence images of common CEnC markers with or without RA in human iPSC line. After day 11 of differentiation, (a-f) CD166 is significantly stronger when RA is used in the induction (a-c) compared to when RA is absent (d-f). (g-l) Tight junction marker ZO-1 shows more polygonal and tightly packed cells with RA (g-i) compared to cell culture without RA (j-l). (m-r) Na^+^/K^+^-ATPase is important for the pump activity and the expression was higher in cell culture with RA (m-o) compared to cell culture without RA (p-r). Data conducted with WT001.TAU.bB2 hiPSC line. Scale bar (a-r) 200 µm.

**Figure S6** Expression of common POM markers in the differentiated cell culture in human iPSC line. After 11 days of differentiation, (a-c) PITX2 expression is stronger in the thick mesenchyme-like structures. (d-f) FOXC1 can be seen in the same structures as well. (g) Phase contrast microscope image of the differentiated human iPSC-CEnC-like cells. Data conducted with WT001.TAU.bB2 hiPSC line. Scale bar (a-g) 200 µm.

**Figure S7** The expression of pluripotency marker decreases totally during the differentiation. (a-c) Fluorescence images of the PSC-CEnCs after 11 days of differentiation shows no expression of OCT3/4 compared to (d-f) undifferentiated cultured pluripotent stem cells. (g) The RT-qPCR analyze shows great decrease of POU5F1 (Oct-4) expression after 3 days of differentiation. Data conducted with Regea 08/017 hESC line. Scale bar (a-f) 200 µm.

**Figure S8** Long RA induction makes some of the cells to form vacuoles and multiple nuclei. Day 6 passaged cells at day 10 with constant 10 µM RA in induction medium made some of the cells to form big vacuoles and huge cells with two or more nuclei. Data conducted with Regea 08/017 hESC line. Scale bar 200 µm.
