# Supplementary figures and images for: Directed Differentiation of Human Pluripotent Stem Cells towards Corneal Endothelial-Like Cells under Defined Conditions
Source: Cells. 2021 Feb 5;10(2):331. doi: 10.3390/cells10020331 (PMC7915025; doi:10.3390/cells10020331)

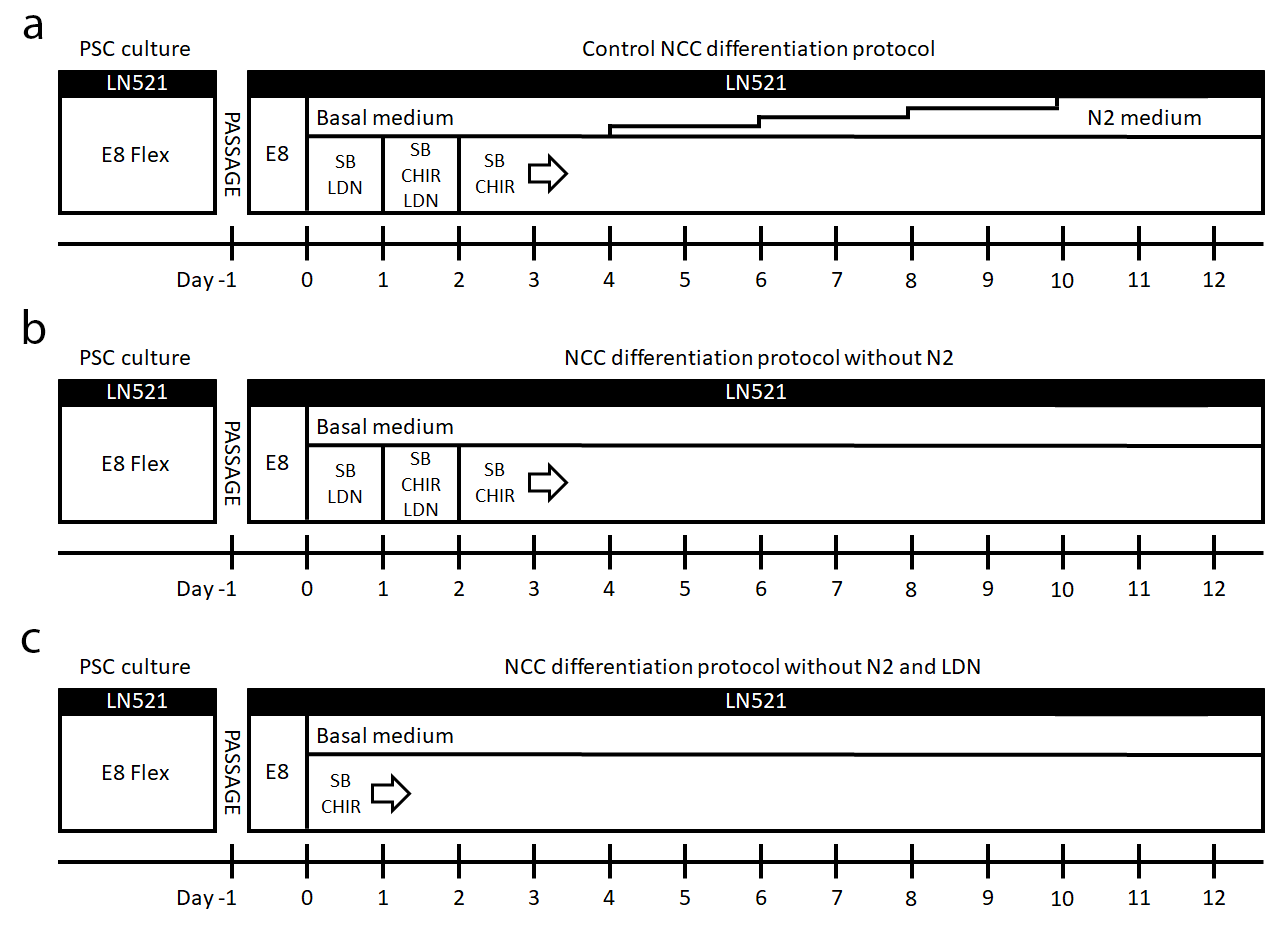

Supplement: Supplementary file 1 [file cells-10-00331-s001.zip › Figure S1.tif]

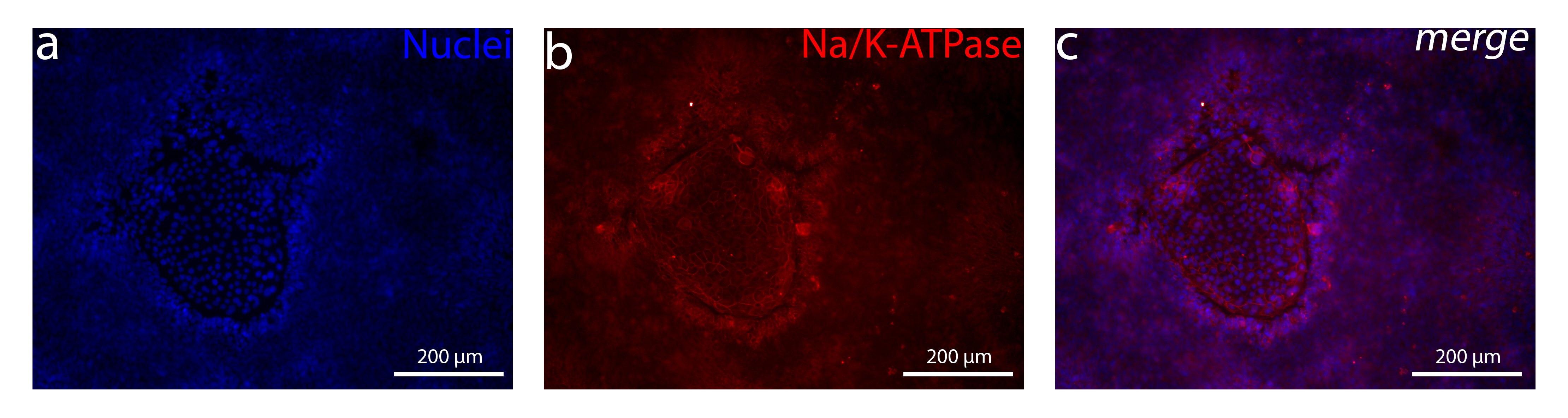

Supplement: Supplementary file 1 [file cells-10-00331-s001.zip › Figure S2.tif]

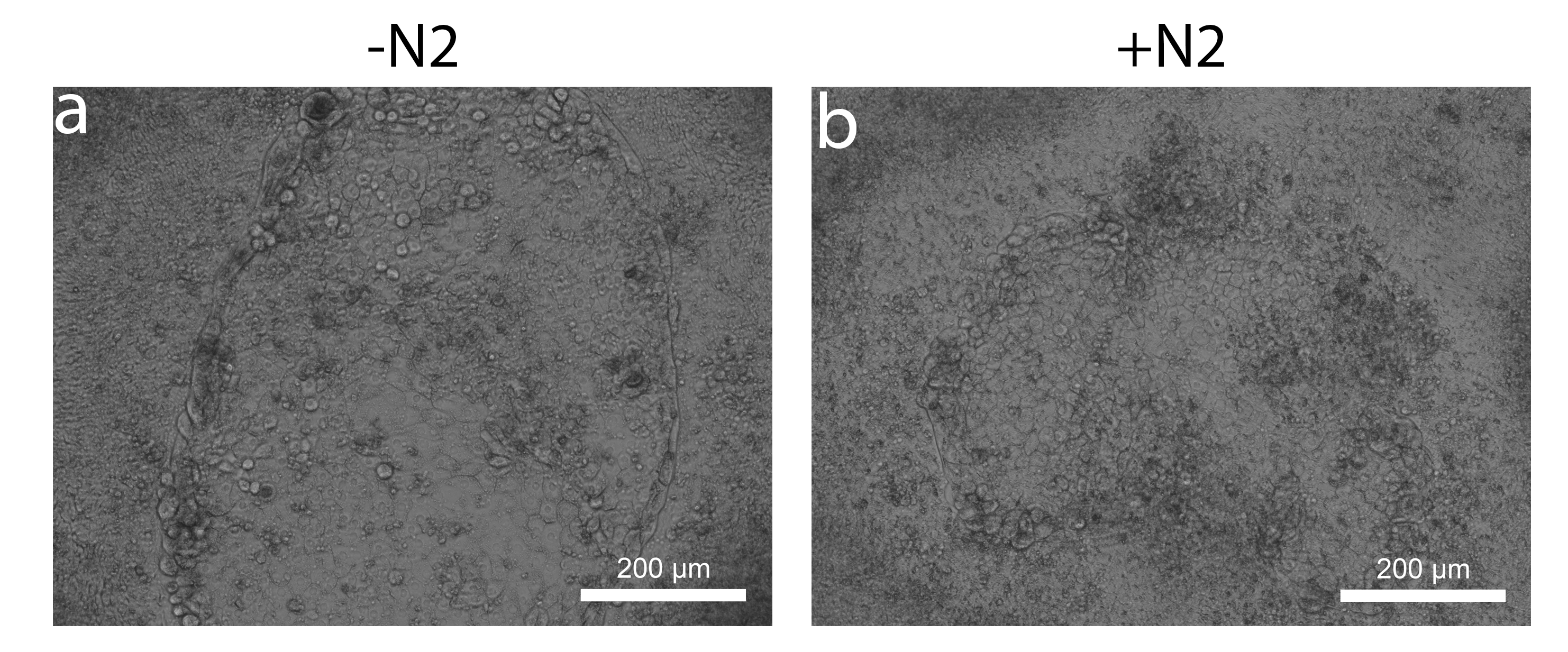

Supplement: Supplementary file 1 [file cells-10-00331-s001.zip › Figure S3.tif]

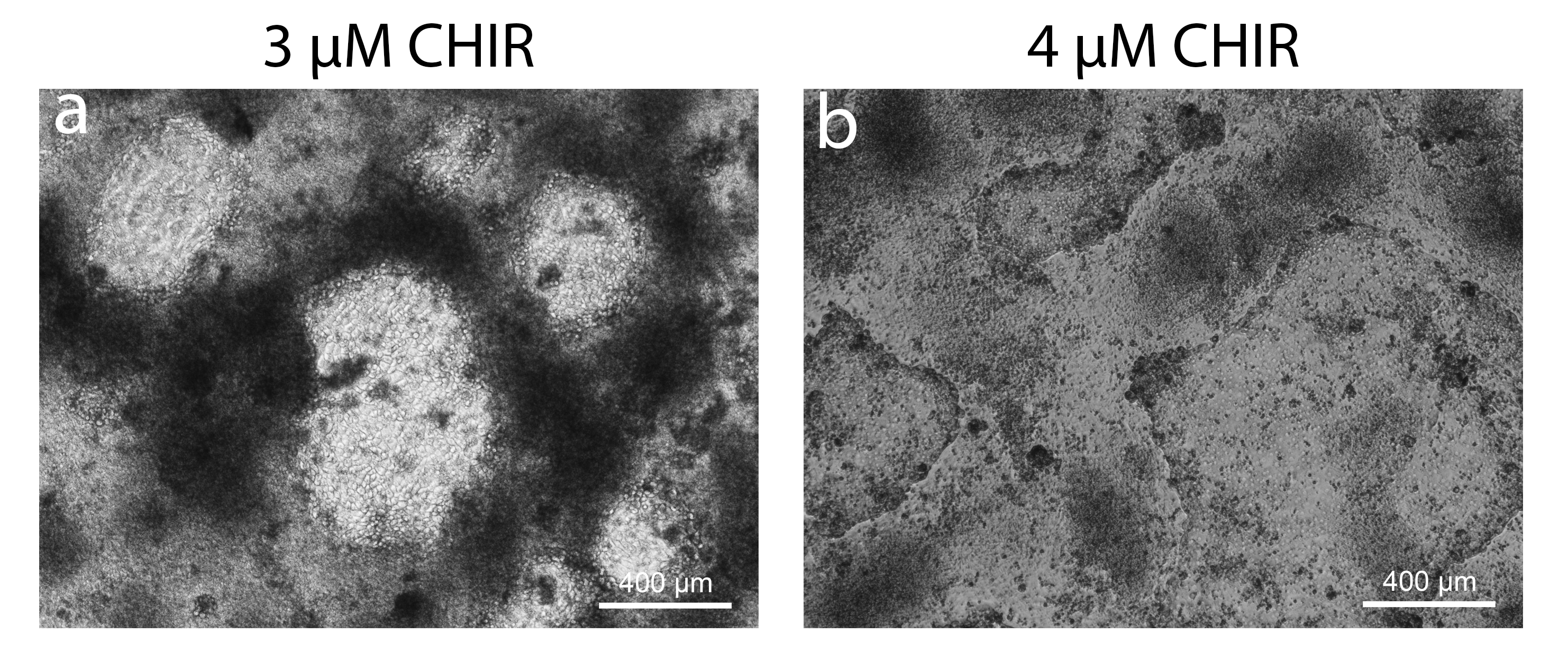

Supplement: Supplementary file 1 [file cells-10-00331-s001.zip › Figure S4.tif]

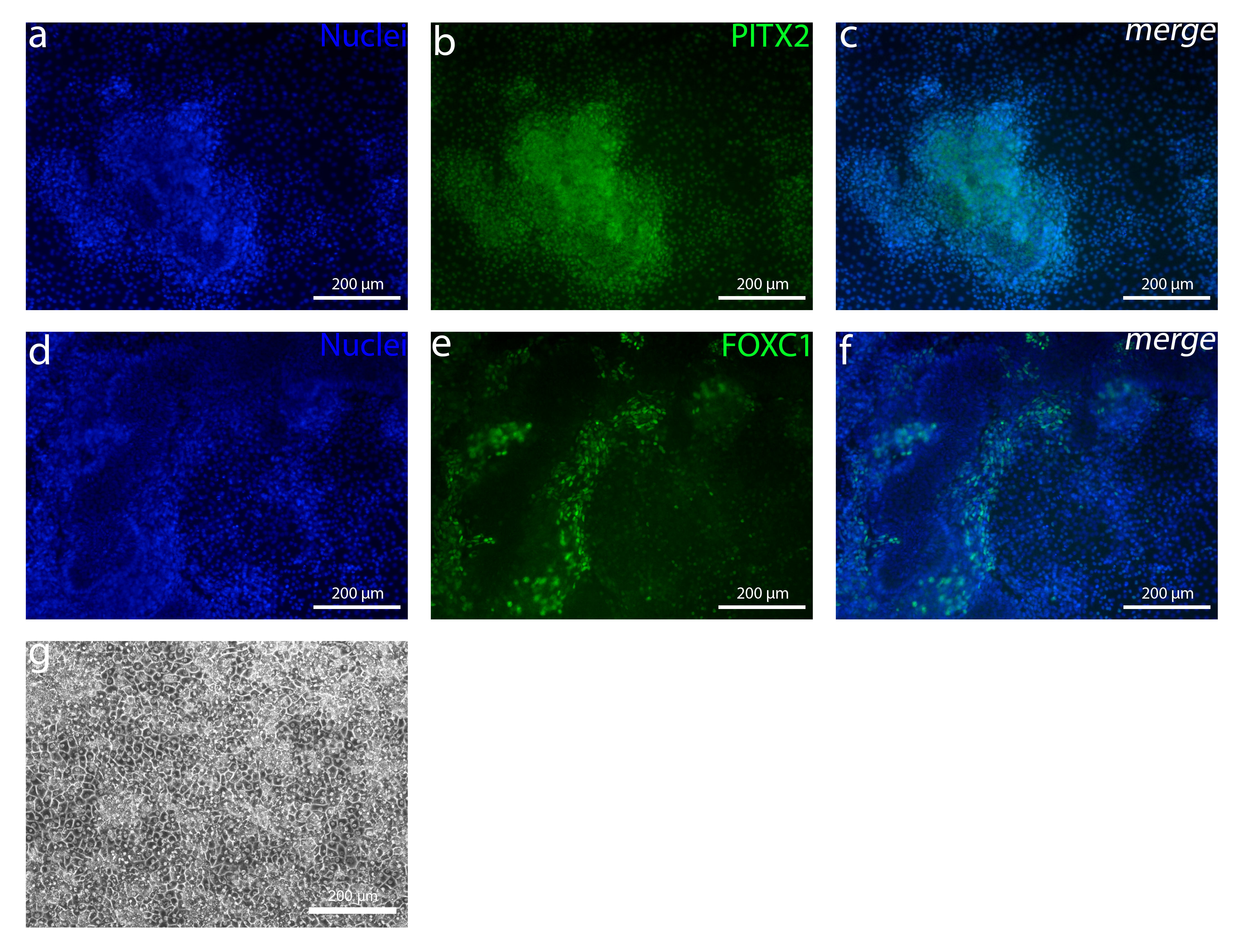

Supplement: Supplementary file 1 [file cells-10-00331-s001.zip › Figure S6.tif]

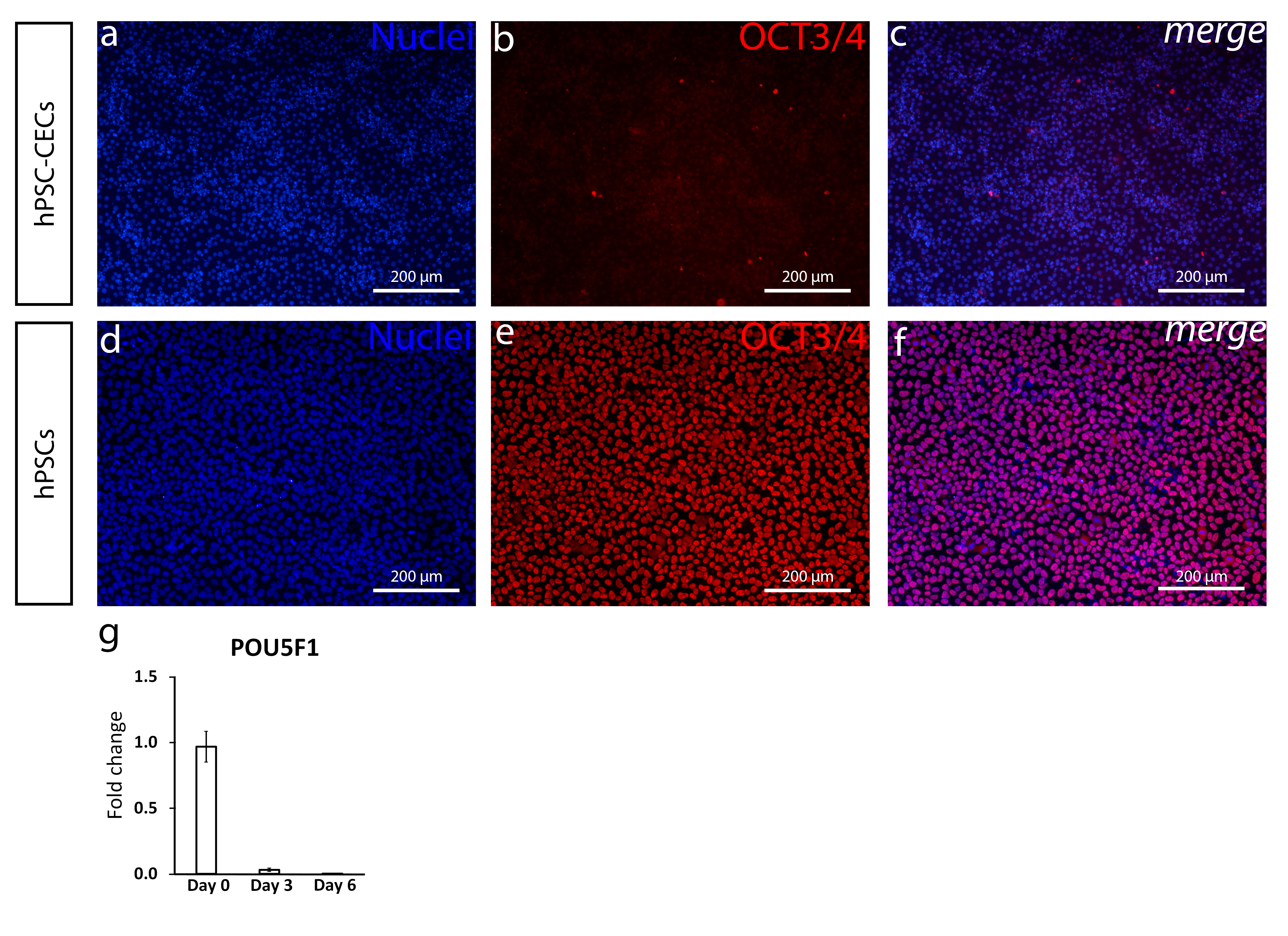

Supplement: Supplementary file 1 [file cells-10-00331-s001.zip › Figure S7.tif]

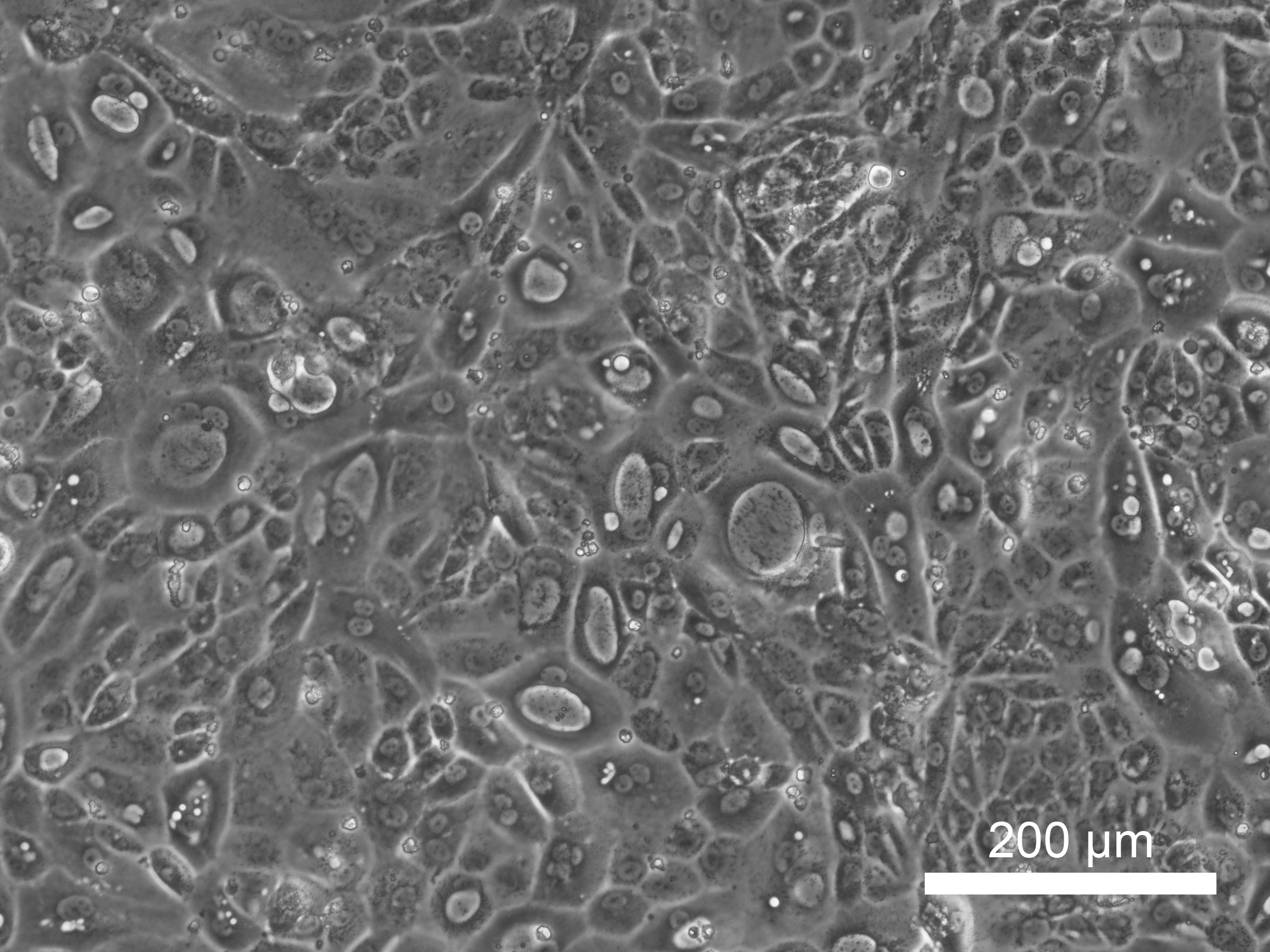

Supplement: Supplementary file 1 [file cells-10-00331-s001.zip › Figure S8.tif]
